# Trigger Finger Appearing as Gradually Increasing Digital Nerve Disorder after Surgical Treatment

**DOI:** 10.1155/2013/542965

**Published:** 2013-03-24

**Authors:** Hiroyuki Tsuchie, Tomio Nishi, Hidekazu Abe, Masaaki Takeshima, Yoichi Shimada

**Affiliations:** ^1^Ugo Municipal Hospital, 44-5 Otomichi, Nishimonai, Ugo 012-1131, Japan; ^2^Department of Orthopedic Surgery, Akita University Graduate School of Medicine, 1-1-1 Hondo, Akita 010-8543, Japan

## Abstract

Trigger finger is a common disease, and operative treatments are often applied for it. Digital nerve injury is one of the complications of this surgical treatment, and paresthesia and sensory disturbance occur early after the operation. This paper presents a case of trigger finger appearing gradually as increasing digital nerve disorder after surgical treatment. In the second surgery, scar tissue covered the palmar MP joint where the A1 pulley had existed before, and palmar digital neurovascular tissue of the ulnar side was found on the inside of the scar. The ulnar digital nerve showed swelling like a neuroma, and bilateral digital nerves existed nearer to the center of the flexor pollicis longus tendon than normal digital nerves. Even when we operate on trigger finger by open release, we should create an appropriate surgical space for observation and be careful of digital nerve injury.

## 1. Introduction

Trigger finger is a common disease, and conservative and operative treatments are often applied for it. Digital nerve injury is one of the complications of this surgical treatment, and paresthesia and sensory disturbance occur early after the operation. We describe herein a unique case of trigger finger appearing gradually as increasing digital nerve disorder after surgical treatment, along with a review of the literature.

## 2. Case Presentation

A 66-year-old man presented at our outpatient clinic with snapping phenomenon of the left thumb for the past 2 years. There was a surgical history of trigger finger of the right thumb, as well as the middle and little fingers. He had undergone two operations for the right thumb because of recurrence. Physical examinations demonstrated mild tenderness over the A1 pulley and snapping phenomenon on finger extension, with no numbness of the thumb. Trigger finger was suspected, and open trigger release was performed. The skin was incised over the A1 pulley. We incised the swelling A1 pulley longitudinally and confirmed the disappearance of snapping of the flexor tendon. Proper palmar digital nerves were not shown. 

After the operation, light paresthesia, not sensory disturbance, of the whole left thumb appeared. This paresthesia of the radial side gradually decreased, but that of the ulnar side gradually increased. At the followup 4 weeks postoperatively, sensory disturbance of the ulnar side appeared. Physical examinations demonstrated a Tinel-like sign near the metacarpophalangeal (MP) joint of the palmar and ulnar side. Although snapping phenomenon on finger extension disappeared, tenderness over the A1 pulley persisted. A second surgery was performed 5 weeks after the first operation.

**Figure 1:**
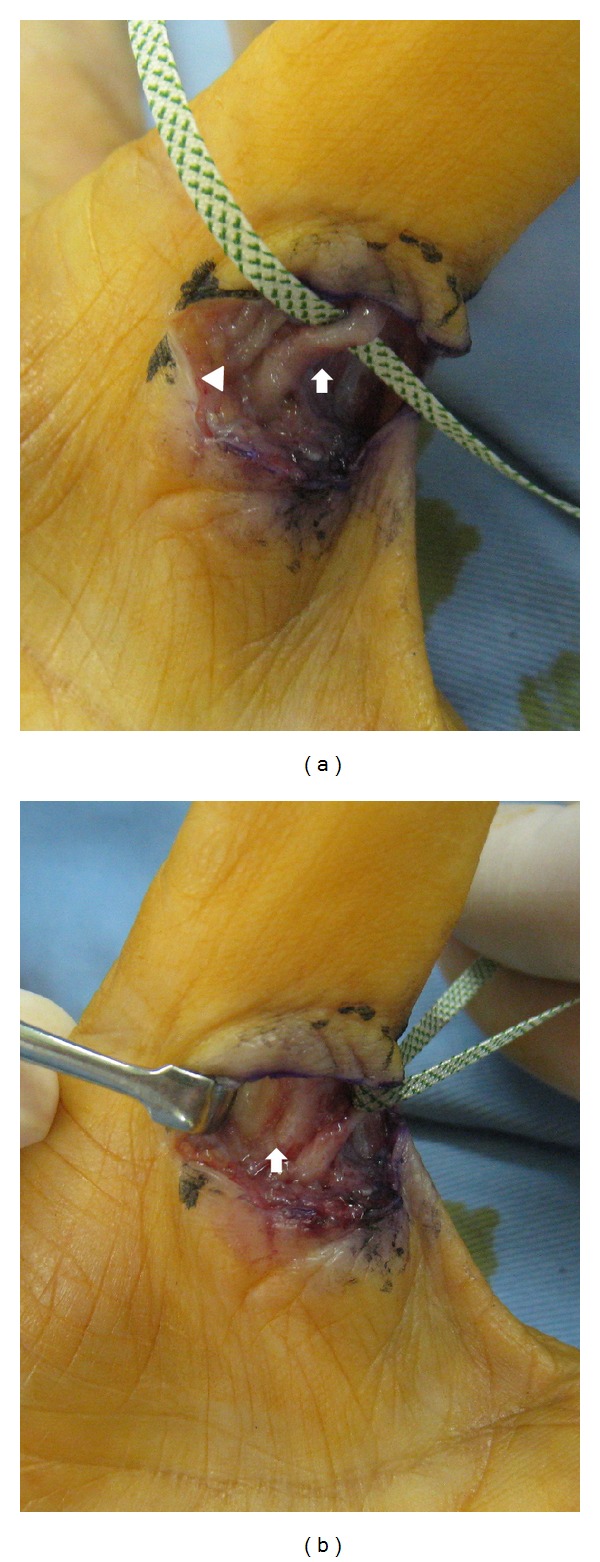
Intraoperative photograph of second surgery. The ulnar (arrow) and radial (arrowhead) neurovascular tissues were located over the tendon and near to the center of it. The ulnar digital nerve showed swelling like a neuroma (a). The flexor pollicis longus tendon was covered by scar tissue (arrow) (b).

We could not find palmar digital neurovascular tissue around the MP joint of the palmar and ulnar side, where we found only adductor pollicis muscle. Scar tissue covered the palmar MP joint where the A1 pulley had existed before. We desquamated this scar, and palmar digital neurovascular tissue of the ulnar side was found on the inside of the scar. Palmar digital neurovascular tissue of the radial side was present on the radial side of this scar, which was not involved in the scar. The ulnar digital nerve showed swelling like a neuroma (Figures 1(a) and 1(b)). We removed this scar, and the flexor pollicis longus tendon appeared under the scar. The ulnar and radial neurovascular tissue was located over the tendon and near to the center of it more than usual. Although we desquamated the distal and proximal neurovascular tissue, they all existed near the center.

Paresthesia and sensory disturbance improved early after surgery, and they were gradually decreased. Tenderness over the A1 pulley was also decreased. The symptoms had almost disappeared two months after the second surgery.

## 3. Discussion

There are some complications of surgical treatment for trigger finger, such as flexor tendon injury, digital nerve injury, digital vascular injury, scar contracture, bowstringing, and complex regional pain syndrome (CRPS) [1, 2]. However, open trigger finger release is generally a safe surgical procedure with a low complication rate [3]. Although the radial digital nerve of the thumb has been described to be the most risky digital nerve during trigger finger release surgery [4, 5], digital nerve injury as a complication was not even encountered in some series [3, 6]. However, once it occurs, it causes a significant morbidity.

In the current case, bilateral digital nerves existed nearer to the center of the flexor pollicis longus tendon than normal digital nerves. So, we assume that bilateral paresthesia of the thumb after the first surgery was caused by the traction of digital nerves in the operation. In addition, we consider that paresthesia and sensory disturbance gradually became worse because increasing scar tissue involved the ulnar digital nerve which exists near to the center of the flexor tendon. Percutaneous surgery for trigger finger has been performed in recent years [7, 8]. If we operated on this case, with neurovascular tissue located near the center of the flexor tendon, using percutaneous surgery, digital nerve injury may occur.

In the current case, the ulnar digital nerve covered with scar tissue showed swelling like a neuroma. Although there is a paper of a case with a neuroma due to injuring the digital nerve after open release of trigger finger [9], there is no report like our case. Some cases with a complication of digital nerve injury may involve the digital nerve due to scar tissue like our case.

In conclusion, the current case is a rare case of trigger finger appearing as gradually increasing digital nerve disorder after surgical treatment. Even when we operate on trigger finger by open release, we should create an appropriate surgical space for observation and be careful for digital nerve injury.
